# The Couch in a Cosey Room

**Published:** 1892-12

**Authors:** 


### THE COUCH IN A COSEY ROOM.

A room without a couch of some sort is only half furnished. Life is full of ups and downs, and all that saves the sanity of the mentally jaded and physically exhausted fortune fighter is the periodical good cry and the momentary loss of consciousness on the upstairs lounge, or the old sofa in the sitting room. There are times when so many of the things that distract us could be straightened out, and the way made clear if one only had a long, comfortable couch on whose soft bosom he could throw himself, boots and brains, stretch his weary frame, unmindful of tidies and tapestry, close his tired eyes, relax the tension of his muscles, and give his harrassed mind a chance. Ten minutes of this soothing narcotic, when the head throbs, the soul yearns for endless, dreamless, eternal rest, would make the vision clear, the nerves steady, the heart light, and the star of hope shine again.

There is not a doubt that the longing to die is mistaken for the need of a nap. Instead of the immortality of the soul business men and working women want regular and systematic doses of dozing—and after a mossy bank in the shade of an old oak that succeeding seasons have converted into a tenement of song birds, there is nothing that can approach a big sofa, or a low long couch placed in the corner where tired nature can turn her face to the wall and sleep and doze away the gloom.

